# Innovative Eco-Friendly Conductive Ink Based on Carbonized Lignin for the Production of Flexible and Stretchable Bio-Sensors

**DOI:** 10.3390/nano11123428

**Published:** 2021-12-17

**Authors:** Daniele Zappi, Gabriele Varani, Enrico Cozzoni, Igor Iatsunskyi, Serena Laschi, Maria Teresa Giardi

**Affiliations:** 1 Biosensor Srl, Via Degli Olmetti 44, Formello, 00060 Rome, Italy; g.varani@biosensor.it (G.V.); mt.giardi@biosensor.it (M.T.G.); 2 Istituto di Cristallografia, CNR Area della Ricerca di Roma, Monterotondo Scalo, 00015 Rome, Italy; 3 Grado Zero Innovation Srl, Via Nove 2/A, Montelupo Fiorentino, 50056 Florence, Italy; enrico.cozzoni@gradozero.eu; 4 NanoBioMedical Centre, Adam Mickiewicz University, Wszechnicy Piastowskiej 3, 61-614 Poznan, Poland; yatsunskiy@gmail.com; 5 Laboratorio Congiunto “Nanobiosens”, Laboratorio di Chimica Medica, Dipartimento di Scienze Biomediche, Sperimentali e Cliniche, Università degli Studi di Firenze, Viale Pieraccini 6, 50139 Firenze, Italy; serena.laschi@ebsr.it

**Keywords:** waste recycling, carbonized lignin, conductive ink, screen-printed electrodes, flexible stretchable bio-sensor

## Abstract

In this study, we report a novel way to produce carbon-based conductive inks for electronic and sensor technology applications. Carbonized lignin, obtained from the waste products of the *Eucalyptus globulus* tree paper industry, was used to produce a stable conductive ink. To this end, liquid-phase compositions were tested with different amounts of carbonized lignin powder to obtain an ink with optimal conductivity and rheological properties for different possible uses. The combination that showed the best properties, both regarding electrochemical properties and green compatibility of the materials employed, was cyclohexanone/cellulose acetate/carbonized lignin 5% (*w/w*), which was used to produce screen-printed electrodes. The electrodes were characterized from a structural and electrochemical point of view, resulting in an electrochemically active area of 0.1813 cm^2^, compared to the electrochemically active area of 0.1420 cm^2^ obtained by employing geometrically similar petroleum-based screen-printed electrodes and, finally, their performance was demonstrated for the quantification of uric acid, with a limit of detection of 0.3 µM, and their biocompatibility was assessed by testing it with the laccase enzyme and achieving a limit of detection of 2.01 µM for catechol as the substrate. The results suggest that the developed ink could be of great use in both sensor and electronic industries, reducing the overall ecological impact of traditionally used petroleum-based inks.

## 1. Introduction

The market for printable inks for electronic applications has considerably grown in recent years. What has been assessed as a market totaling $3 billion in 2020 is expected to reach $4 billion by the end of 2025 [1,2,3].

As of today, the most commonly used inks are silver- or copper-based ones, followed by inks produced using petroleum derivatives such as carbon nanotubes, glassy carbon, and graphene inks [2,4,5].

To minimize the ecological impact of ink production, recent research has been oriented to finding materials from recycling and/or biological sources which, after proper processing, could substitute the carbon from petroleum in the ink preparation. To this end, various materials from different sources have been tried, such as biochar [6,7,8] and carbon black [9].

A material that in recent years has gained the attention of the research community in the development of inks for sensor applications is lignin. The term “lignin” indicates a class of polymeric compounds that have a role as a support tissue in most plants [10]. As opposed to cellulose, lignin is not useful to produce most wood-derivatives, such as paper, and the waste liquid containing a high concentration of lignin (known by the name of “black liquor”) is usually burned on-site for energy production [11,12]. While the majority of the research in electrochemical applications of lignin is focused on its use as a material to build supercapacitors and components for lithium batteries [13,14,15,16], the focus is now shifting to the production of high-quality electrodes for precision electrochemical determinations, using different techniques to transform lignin into a suitable material, such as laser-induced graphitization [17] and nitrogen-doped laser-scribed graphene [18].

One further procedure that can be employed to transform lignin into a conductive material is carbonization: lignin is placed in an oxygen-deprived chamber and heated to temperatures ranging from 600 °C to 1300 °C. The physic-chemical characteristics of the obtained material, as well as its chemical structure, depend on the temperature applied [19]; this procedure has been used on lignin obtained from different industrial processes [20], examining how the different industrial processes yield lignin with different structures and how the carbonization process affects their overall physic-chemical properties. Lignin carbonization was also researched to produce high-quality nanostructured materials such as carbon black [21].

In this work, several materials, refined from the wastes of the paper industry cycle, have been tested in different proportions to create a conductive, carbon-based ink having a conductivity and rheological properties optimal for screen-printing. The innovative developed ink made it possible to produce screen-printed electrodes (SPEs) on a semi-industrial manufacturing level and quantity. Furthermore, the electrodes produced have noticeable flexible and stretchable properties. The electrochemical properties of the produced electrodes were assessed by different analytical techniques. Furthermore, the conductivity properties of the electrodes when undergoing single and cyclical stress tests at different elongations were tested to evaluate their suitability as wearable sensors. To demonstrate the applicability of the developed SPEs as support platforms for sensors, a sensor for uric acid was developed and characterized to analyze if the SPEs suffered from fouling phenomena due to the formation of a strong interaction with electrochemical products compared to the electrodes produced employing commercial inks. Furthermore, the SPEs were tested with the laccase enzyme, to assess the carbonized lignin-based ink’s compatibility for future biosensor development.

These newly developed SPEs could result in a decrease of the environmental impact of platforms in which conductive inks are used for in-field medical analysis [22,23,24,25], the agrifood industry [26,27], water quality monitoring [28,29], and tracks for integrated circuit boards in systems that require both a high conductivity and flexibility [30].

## 2. Materials and Methods

### 2.1. Materials

Catechol, uric acid, tetrahydrofuran (THF), cyclohexanone (CEx) cellulose acetate (CA), polyurethane (PU), glycerol, sodium chloride, and laccase enzyme from *Trametes versicolor* (code 53739-1g) were purchased from Sigma-Aldrich (Merck KGaA, Darmstadt, Germany), ethanol 96% (*v/v*), AnalaR NORMAPUR^®^ analytical reagent was purchased from VWR Chemicals (Milan, Italy). Milli-Q water was used in all experiments. Polyethylene terephthalate (PET), polyurethane (PU), and PET/PU composite sheets were provided by Policrom Screens S.P.A. (Carvico, BG, Italy). The silver reference electrodes and contacts on all the produced electrodes were printed using LOCTITE^®^ ECI 1010 E&C (Henkel AG & Co., KGaA, Düsseldorf, Germany); the working and reference electrodes of the commercial electrodes were printed using LOCTITE^®^ EDAG 407C E&C (Henkel AG & Co. KGaA).

### 2.2. Carbonized Lignin Production

Lignin with low sulfonate content, produced through the Kraft process from the Eucalyptus tree, was used as the starting material. Before carbonization, the material was dried in an oven at 40 °C for 12 h. The material was then ground in a planetary ball mill (type: Retsch PM100) with four 40 mm diameter balls at 250 rpm for 2 h, with counter-rotation occurring after 1 h to reduce the size of the large particles, to a powder consistency. The lignin was then placed in a combustion vessel and inserted into the center of a horizontal tube furnace.

Preliminary thermal stabilization was obtained at 250 °C, in an ambient atmosphere, to avoid fusing the particles because of the inherent thermoplasticity of lignin during carbonization [31,32]. The mass loss resulting from this treatment was 35% mass loss.

During the thermostabilization, several processes contributed to the mass loss. First, the initial mass loss was caused by the removal of residual moisture. Thereafter, the oxidation of lignin caused a further mass loss that was associated with the loss of phenolic, alcoholic, methyl, carbonyl, and methoxyl groups [31,32]. While these phenomena primarily led to a loss of oxygen, hydrogen, and carbon, the inorganic compounds were not affected.

The carbonization treatment under an argon atmosphere, which followed thermostabilization, caused the remarkable additional mass loss. For the lignin, mass loss was observed after the first two carbonization steps with the target temperatures of 800 °C and 1200 °C, whereas only minor mass changes occurred at the higher temperatures.

During the carbonization, the tube was sealed at both ends, and argon gas was flushed through the tube to remove any oxygen to attain pyrolysis conditions. With a continual argon gas flow through the tube, the furnace was set to a heating rate of 20 °C min^−1^ until the carbonization temperature was reached and remained isothermal at the given temperature for 8 h before cooling to room temperature under argon flow. The carbonized material was removed from the furnace upon reaching room temperature. Structural changes in lignin being correlated to the carbonization temperature are well-documented in the literature [19]. Since we aimed to obtain material that is as conductive as possible, the carbonization temperature corresponding to the highest degree of conjugated bond formation was chosen, that is, 1200 °C.

After the identification of the optimized temperature of the carbonization process, the carbonized material was milled in the planetary ball mill (type: Retsch PM100). From the literature [21] it is known that a 24 h milling period results in the maximum increase of the surface area of the carbonized material. The material was used to produce the ink with a high surface-to-mass area, in order to maximize both the solubility in the ink solvent and, once dried, the surface area to interact with analytes during electrochemical analyses. Thus, a ball milling time of 24 h was selected as it was reported as producing the highest surface area.

The 24 h ball milled carbonized lignin powder, carbonized at 1200 °C, was used in all subsequent experiments.

### 2.3. Ink Preparation

To prepare ink suitable for screen-printing, 0.05 g of cellulose acetate were dissolved in 1.5 mL of cyclohexanone, then carbonized lignin in the proportion of 5% *w/w* with the solvent was added. The resulting mixture was sonicated in a closed container to prevent solvent evaporation for 1 h. The obtained suspension was stable for days but, in every case, was used immediately after production.

### 2.4. Electrode Printing

For electrode production, the screen-printing process was employed. Silver traces and reference electrodes were printed using a commercial silver ink spread over a 120 threads/cm frame. For the silver traces, a sinusoidal shape was adopted since it has been proved in the literature [33] that such shapes show lower resistance changes when undergoing mechanical stress. Two passes were performed to obtain good ink coverage. After printing, ink was polymerized in an oven at 65 °C for 12 h. Commercial carbon ink was printed by spreading it over a 70 threads/cm frame. After printing, ink was polymerized in the oven at 65 °C for 12 h. Ink obtained from carbonized lignin was firstly passed into a roller homogenizer mill subjecting it to 10 consecutive passes (maximum number established experimentally as a compromise between a good homogenization and the solvent evaporation speed). The homogenized ink was printed by spreading it over a 70 threads/cm frame. After printing, the ink was polymerized in the oven at 65 °C for 12 h.

### 2.5. Scanning Electron Microscopy and Raman Spectroscopy

To study the morphological and chemical properties of the produced electrodes, scanning electron microscopy (SEM) with an energy dispersive X-ray analysis (EDX) and Raman spectroscopy were used. The surface morphology was investigated by a SEM JEOL JSM-7001F (operating voltage 15 kV) with an EDX analyzer (Oxford Instruments XMax 80 mm^2^ detector). Raman spectroscopy was performed using a Renishaw micro-Raman spectrometer equipped with a confocal microscope (Leica, Wetzlar, Germany). The samples were measured in a backscattering geometry with a spectral resolution better than 1.0 cm^−1^. The incident light was not polarized and also the light detector contained no polarization filters. The Raman scattering spectra were excited by a 633 nm laser. The beam was focused on the samples with a 50× microscope objective with a numerical aperture of 0.4.

### 2.6. Electrode Stretching Tests

Mechanical tests were performed to assess the elasticity of the electrodes and their electrical properties at different elongations. Elasticity and flexibility are fundamental properties for wide use electrodes and to allow the creation of miniaturized devices. The electrode should maintain the electrical properties within reasonable variations for short as well longer elongations (for instance, an increase of length greater than 25% of the original length, which is a possible elongation in the field of wearable devices). For this purpose, a simple mechanical tool was used to perform the stretch test of the electrodes. This tool can lock the stretchable electrode from one side and hold on to the position. The opposite end can then be stretched and locked at different elongation percentages, which are reported on the side of the instrument.

### 2.7. Working Electrode Surface Activation

The surface of the working electrodes of the produced SPEs needed to be activated before modification to produce biosensors or to use them in electrochemical measurements. To this end, different activation techniques were tested and the results obtained were compared with those obtained by applying the same techniques to a commercially produced electrode. The activation procedures should increase the porosity of the working electrode, thus creating a 3D structure, which is more suitable for electrochemical exchanges between an electrode and the solution. Numerous activation procedures are known in the literature [34]; among these, two were tested on the produced SPEs, one depositing a strong acid coupled with an electrochemical treatment, the second depositing a strong oxidizing agent.

The activation procedures entail the following:

(i) Activation with 1 M hydrochloric acid coupled with cyclic voltammetry (CV): A total of 60 µL of 1 M HCl was deposited on the electrode surface, covering the working, reference, and counter electrode. Five CV scans, with a potential range from −1.0 V to +1.0 V and a scan speed of 10 mV s^−1^ were performed. In the end, the electrode was thoroughly washed and allowed to dry.

(ii) Activation with nitric acid: A total of 20 µL of HNO_3_ (at a concentration of 1 M or 2 M) was dropped on the working electrode of the SPE and left to react for 30 min. After, the electrode was thoroughly washed and allowed to dry. Each activation procedure was performed on three independent SPEs, both commercial and produced with the in-house produced ink.

### 2.8. Electrochemical Performances Evaluation

To evaluate the activation effect on the electrode surface, activated and non-activated electrodes were analyzed through cyclic voltammetry by dropping 50 µL of 2.5 mM K_3_Fe(CN)_6_ + 2.5 mM K_4_Fe(CN)_6_ + 0.1 M KCl as the electroactive solution on the electrode (taking care to cover the working, reference and counter electrodes) and performing the cyclic voltammetry scans from −1 V to +1 V, with a scan speed of 50 mV s^−1^.

To further analyze the electrochemical properties of the lignin-based electrodes, cyclic voltammetry measurements at increasing scan speeds were used to calculate the electrochemically active areas (E_a_) for the non-activated lignin-based electrode and HNO_3_-activated lignin electrodes. The results were compared with those obtained using an electrode printed using commercial carbon ink. Using a solution containing an electrochemically active molecule capable of reversible redox reaction (50 µL of 1 mM K_3_Fe(CN)_6_ + 0.1 M KCl), the Randles–Sevcik equation can be applied [35].

Peakintensity=(2.99×105)×n32×Ea×C×D12×v12

where v is the scan speed (V s^−1^); n is the number of electrons involved in the redox process; E_a_ is the electrochemically active area (cm^2^); C is the concentration of the electroactive analyte in solution (mol L^−1^); and D is the diffusion coefficient for the selected electroactive analyte (cm^2^ s^−1^).

The measured peak current (anodic or cathodic) is thus linearly proportional to the electro analyte concentration and proportional to the square root of the scan speed.

### 2.9. Electrochemical Experiments

Measurements of the resistance of inks and electrodes were performed using a Keithley Integra Series 2700 multimeter/data acquisition system.

All electrochemical experiments were conducted using a Uniscan potentiostat (Uniscan PG580RM, Uniscan Instruments Ltd., Buxton, UK).

## 3. Results and Discussion

### 3.1. Ink Preparation and Optimization

To produce inks suitable for the screen-printing process, different preparations were tested using various organic solvents and binders. The first was necessary, since carbonized lignin suspends poorly in water, forming lumps that do not break even when undergoing energetic sonication. To solve this problem, two different solvents were tested: tetrahydrofuran (THF) and cyclohexanone (CEx). At the same time, binders need to be added to the ink mixture, since they are the component that links the conductive material (in our case the carbonized lignin) to the electrode substrate once the solvent evaporates. The binders tested in this work were cellulose acetate (CA), polyurethane (PU), polyvinylchloride, and glycerol.

THF proved suitable to suspend the carbonized lignin powder forming, together with a binder, macroscopically homogeneous emulsions after sonication. Each combination prepared was applied as a thin layer on a PET surface. For each of the different combinations tested, the resistance on the dried composite was measured at a fixed distance. Even though the ink formulations performed well when mechanically spread on the PET substrate, they showed several drawbacks that limited their application to produce flexible and/or stretchable screen-printed electrodes: mainly, all THF-based ink formulations dried too quickly to be suitable for screen-printing at an industrial level; after drying, THF-based inks showed little flexibility, often cracking when undergoing even small flection stress, thus not proving suitable for flexible electrode production; and, finally, THF is highly toxic for humans and the environment, making the large-scale production of electrodes difficult.

To overcome the issues related to the use of THF in electrode production, cyclohexanone was used as an alternative in an equal weight amount. This solvent was chosen as it is largely used in the industrial production of screen-printed electrodes [36,37,38] and it also achieves an overall lower ranking in the “CHEM21 selection guide of classical- and less classical-solvents” [39]. Thus, three new inks were prepared using the previously employed binders, and a new binder, glycerol, was also tested. These inks were then applied on a PET surface but, as CEx dries much slower than THF, a procedure of ink drying was implemented to mimic the one commonly used in SPE industrial production: after applying a uniform layer of ink, the PET substrate was put in an oven and heated at 120 °C for 20 min. All inks showed a good flexibility, meaning no detachment of the ink and/or formation of cracks on the ink surface occurred when the application substrate was subjected to flection stress (up to an angle of 90°). The inks prepared using PU or CA as binders showed a good adhesion to the PET substrate, while the one prepared using glycerol was easily removed from the PET surface. Resistance values were measured after heating; the results obtained along with the adherence and flexibility properties are reported in Table 1.

As the ink using glycerol as a binder showed bad adhesion properties, while the one based on PU showed a too high resistance, the ink based on CA was used for further optimization studies.

### 3.2. Optimization of Carbonized Lignin Content in CA-Based Inks

Formulations with a concentration of carbonized lignin in the range of 5–20% *w/w* were analyzed. Six systems were studied with a carbonized lignin content of 5, 7.5, 10, 12, 15, and 20% *w/w*, respectively.

This interval was chosen considering the viscosity and the attaching properties of the formulation over PET. The ink was sonicated for 3 h to allow for the homogenization and the correct dispersion of the carbon powders with the CA binder. For a concentration of carbonized lignin higher than 20%, the formulation was too viscous to be spread over the support. Moreover, for a concentration higher than 20%, the layer formed after drying was brittle and fragile, thus making the resistive measurements difficult to perform and not suitable for flexible/stretchable electrodes. On the other hand, carbonized lignin concentrations lower than 5% resulted in an ink viscosity that was too low and thus not suitable for the screen-printing technique.

Resistance values obtained for each ink preparation were 77 ± 8 Ω for 5%CL, 57 ± 6 Ω for 7.5%, 27 ± 2 Ω for 10%, 35 ± 3 Ω for 12%, 33 ± 3 Ω for 15%, and 415 ± 50 Ω for 20%.

The carbonized lignin concentrations giving rise to the most interesting results were those ranging from 7.5 to 15%. To perform a more thorough study, the carbon ink on the commercial SPEs printed on a PET substrate was replaced with the different ink formulations produced. To do so, the commercial carbon ink was firstly removed from the WEs and CEs of the commercial electrodes by careful use of CEx. Then, the carbonized lignin-based ink was applied in substitution by using a properly designed mask that had ink-permeable zones corresponding to the position of the WE and CE on the commercial electrode, thus simulating the industrial process used for SPE printing. After ink application, the electrode was dried in an oven at 65 °C for 12 h, to induce uniform ink drying and adhesion to the substrate.

Once the ink was dried, conductivity was measured between the center of the working electrode (or counter electrode) and the relative silver connector. The results obtained are reported in Table 2.

Considering the data presented in Table 2, the best results appear to be those in which carbonized lignin ink at 5% concentration was used to substitute the commercial ink. Even if these results appear to be in contrast with those previously obtained when applying the ink on the PET surface, it can be assumed that inks containing low concentrations of carbonized lignin interface better with the underlying support material.

The formulation giving the best results (CEx/CA/CL 5%) was used to produce screen-printed electrodes on the PET and PET/PU substrates.

### 3.3. Electrode Design and Printing

#### 3.3.1. Electrode Design

**Figure 1:**
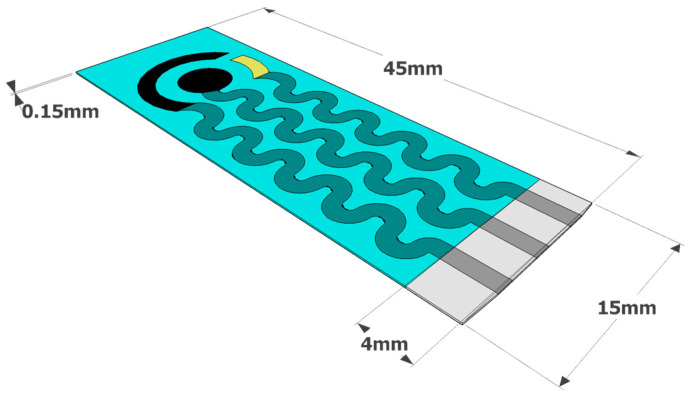
Schematic with dimensions of the design chosen for SPE printing that minimizes electrochemical variation when undergoing mechanical stress (taken from patent [40]).

New screen-printed electrode designs were developed, studied, and tested to evaluate the one with the best performances under physical stress to achieve the best support for stretchable, wearable applications. The final design is reported in Figure 1.

As shown in Figure 1, the working, reference, and counter electrodes were arranged in a classical concentric shape, ideal for electrochemical measurements. On the other hand, the silver tracks between each electrode and the connector to the instrument had a sinusoidal shape, which suffered minimal conductivity variation even when undergoing considerable mechanical stress (as described in Section 3.4).

#### 3.3.2. Screen-Printed Electrode Printing

**Figure 2:**
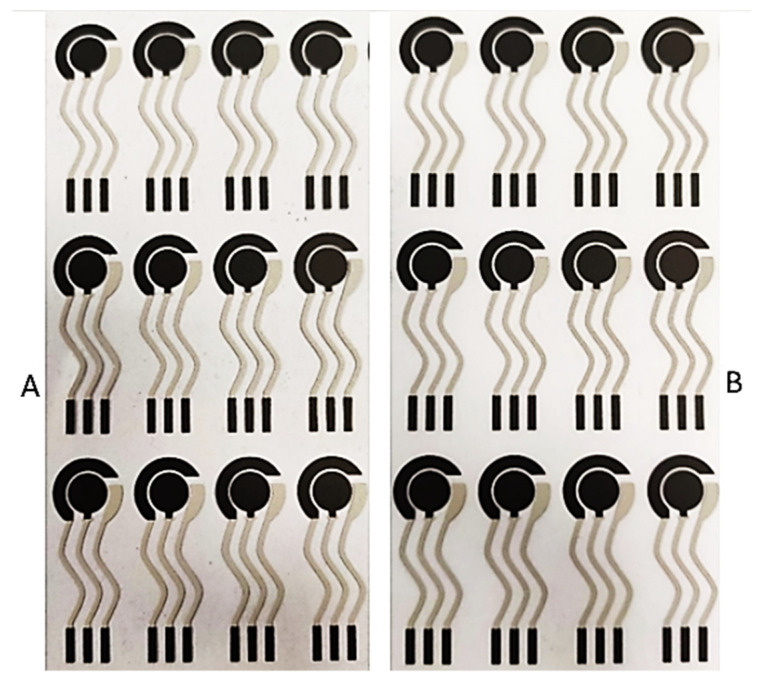
(**A**) Electrodes printed on PET flexible substrate and (**B**) electrodes printed on PET/PU stretchable substrate.

The optimized ink formulation developed in Section 3.3 was used to simulate a semi-industrial production of SPE using the electrode scheme reported in Section 3.3.1, PET sheets of 125 µm thickness (to obtain flexible SPEs) and composite PET/PU sheets of 100 µm thickness (to obtain flexible and stretchable SPEs) were used as the substrate materials. Some examples of the SPE sheets obtained are reported in Figure 2.

There was a particularly good degree of visual repeatability between sheets of printed SPEs, even across different substrate materials. This is further underlined in the detailed photos of the printed electrodes. The ink developed results in a clear print, with both working and counter electrodes well defined and separated both between themselves and from the silver reference electrode.

### 3.4. SEM Analysis and Raman Spectroscopy

**Figure 3:**
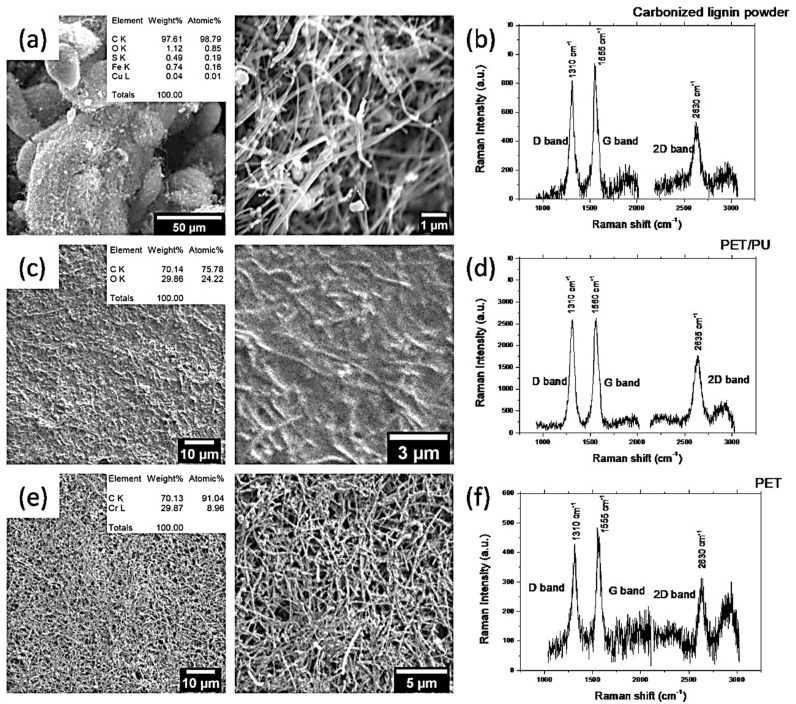
SEM images and Raman spectra for (**a**,**b**) the carbonized lignin powder, (**c**,**d**) PET/PU, and (**e**,**f**) PET electrodes (inset-EDX analysis).

Figure 3 reports the morphology, chemical composition, and Raman spectroscopy of the carbonized lignin powder (Figure 3a,b), the produced carbon PET/PU (Figure 3c,d), and the PET (Figure 3e,f) electrodes. The carbonized lignin powder presented macroparticles with an average size of 10–80 microns consisting of tightly intertwined carbon nanofibers. The average diameter of the carbon nanofibers ranged from 200 to 400 nm. An EDX analysis showed that there was a small amount of metal impurities and oxygen present in the powder. There were three Raman peaks at 1310, 1555, and 2630 cm^−1^ corresponding to the D, G, and 2D peaks of carbon, respectively (Figure 3b). The Raman spectrum corresponds well to the carbon fibers and/or multiwall carbon nanofibers [41]. It should be noted that the behavior of the Raman spectra, and therefore the carbon, remained almost the same for the produced electrodes. However, the SEM analysis of the PET/PU and PET surfaces indicated the difference in both morphology and chemical composition. The surface of the PET/PU substrate appeared to be a composite of fibers/polymer with a high concentration of oxygen. It was challenging to obtain SEM images because of the surface charging effects due to the high polymer concentration. The surface of the PET substrate consisted of carbon fibers with a dense distribution of fibers compared to the initial carbonized lignin powder (Figure 3e). The presence of chromium can be explained by the metallization tracks of the electrodes.

### 3.5. Electrode Stretching Test Results

**Figure 4:**
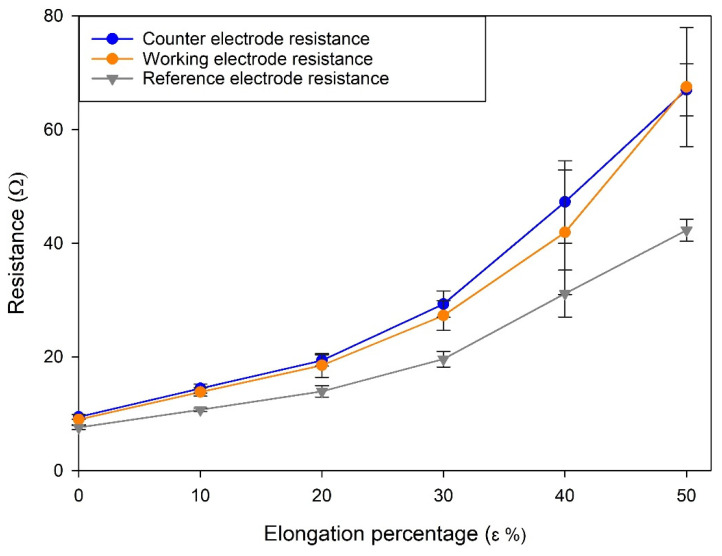
Resistance variation trends at different elongation coefficients. Each line corresponds to a different electrode of the SPE: counter electrode (blue), working electrode (orange), reference electrode (grey).

The effect of mechanical stress on the electrical properties of the stretchable electrodes was studied considering the variation of resistance values of the working, counter, and reference electrodes between the rest and stretched states. Five total stretched states were evaluated, ranging from 10% to 50% as the elongation coefficient, calculated as a percentage increase in sensor length compared to the rest state. Each stretch state was evaluated using at least three different electrodes, the results obtained were averaged, and the standard deviation was calculated. The results obtained for each stretch state are reported in Figure 4.

From the data obtained, a quasi-linear proportionality between the extension coefficient ε and the resistance measured for the three electrodes can be observed up to a 30% elongation (R^2^ = 0.97). This can be interpreted as a sign of the good elastic response of the electrode platform and a small deterioration of both the electrodes themselves and the silver tracks used to connect them to the instrument. For elongation coefficients higher than 30%, a sharp rise of measured resistance was observed. This, coupled with the higher standard deviation of the resistance values obtained, suggests that at these stretch levels the electrode structure undergoes irreversible stretching, which results in damage occurring to the electrodes and/or to the silver tracks. It can be concluded that the proposed stretchable electrodes can be used for length increase up to 30% of the original length without suffering significant degeneration.

**Figure 5:**
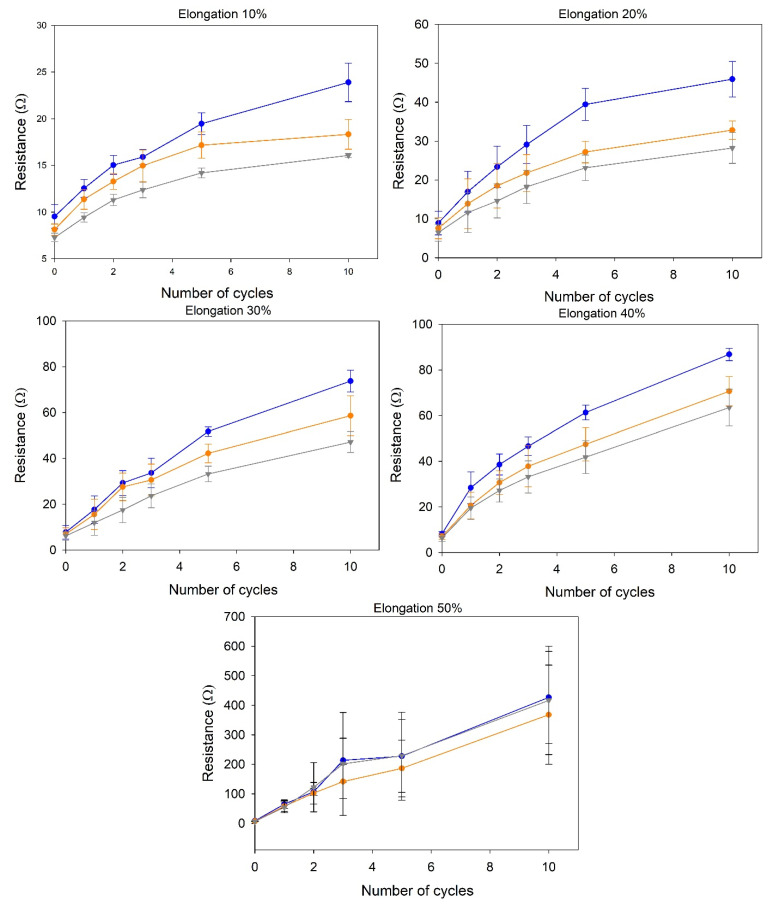
Different resistance variation trends for repeated stretching at different elongation percentages. Each line corresponds to a different electrode of the SPE: counter electrode (blue), working electrode (orange), and reference electrode (grey).

A second test was performed to study how multiple cycles of mechanical stress (at a fixed stretch length) followed by relaxation affected the resistance value for the three electrodes (loading/unloading stress). This study was performed to simulate the stress affecting the stretchable electrodes during normal use, for example, when integrated into clothes. Once again, five total stretched states were evaluated, ranging from 10% to 50% stretching, calculated as a percentage increase in sensor length compared to the rest state. For each stretch state, up to 10 cycles of stretching were performed, and the resistance values of the working, counter, and reference electrodes were measured at the end of each cycle. Each stretch cycle was evaluated in triplicate, the results obtained were averaged, and the standard deviation was calculated. The results obtained for each stretch state are reported in Figure 5.

A common trend can be seen for all graphs, which is an increase in the measured resistance correlated with the number of stretching cycles performed. It should be underlined that, for small elongation percentages (10% and 20%), the resistance increase reached a plateau value at around 10 stretching cycles, indicating that further stretching cycles would alter the overall electrode resistance in a small way. This behavior did not apply for longer elongations (30% and over), where the resistance increase followed an almost linear correlation with the number of cycles. It can be concluded that the developed electrode can be integrated into textiles that can be subjected to small to moderate stretching, i.e., the lower back. The stretchable electrodes produced using the commercial ink for the WE demonstrated similar trends to the ones shown in Figure 4 and Figure 5 (data not shown). It can be assumed that, since both types of electrodes employ the same silver ink for the sinusoidal connections (Figure 1), they undergo similar deformations when mechanical stress is applied.

### 3.6. Carbonized Lignin SPE Activation Results and Comparison with SPE Produced Using Commercial Inks

**Figure 6:**
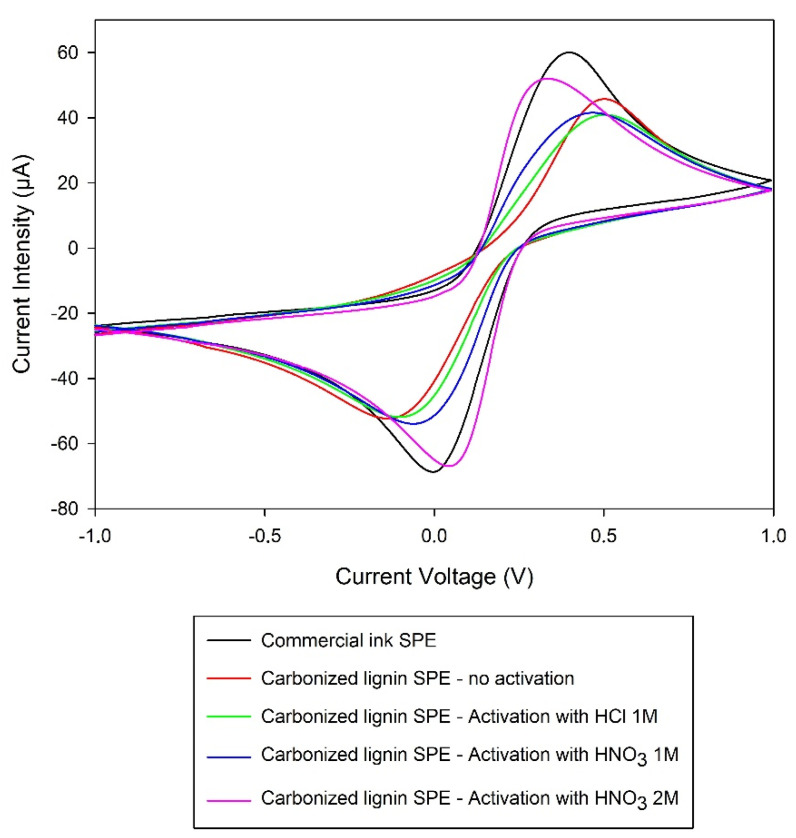
Comparison of CV results obtained with carbonized lignin electrodes activated with different procedures. Measurements performed using a solution of 2.5 mM K_3_Fe(CN)_6_ + 2.5 mM K_4_Fe(CN)_6_ + 0.1 M KCl (other experimental conditions are reported in Section 2.8).

To evaluate if the SPEs produced with the carbonized lignin-derived ink in substitution of the commercial carbon inks have satisfying electrochemical properties, electrochemical analyses were performed on the unmodified and activated carbonized lignin electrodes (as described in Section 2.8) and compared with the results obtained using SPEs having a similar working electrode diameter produced using commercial carbon inks. An example of the results obtained is reported in Figure 6.

As reported in Figure 6, the activation procedures involving the use of 1 M HCl or 1 M HNO_3_ (green and blue curves, respectively) did not give significantly different results from those obtained using the non-activated electrode (red curve). On the other hand, the activation of carbonized lignin electrodes using 2 M HNO_3_ resulted in performances remarkably similar to the ones obtained employing the electrode produced with commercial inks. The distance between the oxidation and reduction peaks, which is a good index of the reversibility of the process happening on the electrode, was calculated as 0.402 V for the electrode made with commercial ink, 0.638 V for the non-activated CL-based electrode, 0.599 V for the CL-based electrode activated using the HCl procedure, 0.532 V for the CL-based electrode activated using 1 M HNO_3_, and 0.380 V for the CL-based electrode activated using 2 M HNO_3_. Thus, the activation procedure using 2 M HNO_3_ was employed on all the electrodes used in the subsequent tests and applications.

### 3.7. Electrochemically Active Area Evaluation

As shown in Section 2.8, the Randles–Sevcik equation justifies a linear relationship in the cyclic voltammetry of a redox reaction between the peak intensity (anodic or cathodic) and the square root of the scan speed (v) used. As the parameters n (number of electron exchanged = 1), C (concentration of the electroactive substance), and D (diffusion coefficient = 7.6 × 10^−6^ cm^2^/s) are known, it is possible to rearrange the equation as

m=(2.99×105)×n32×Ea×C×D12Peakintensity=m×v12

Thus, by plotting the intensity of the peak against the square root of the scan speeds employed in the analysis, it is possible to calculate via linear regression the coefficient m and, thus, the value of E_a_.

**Figure 7:**
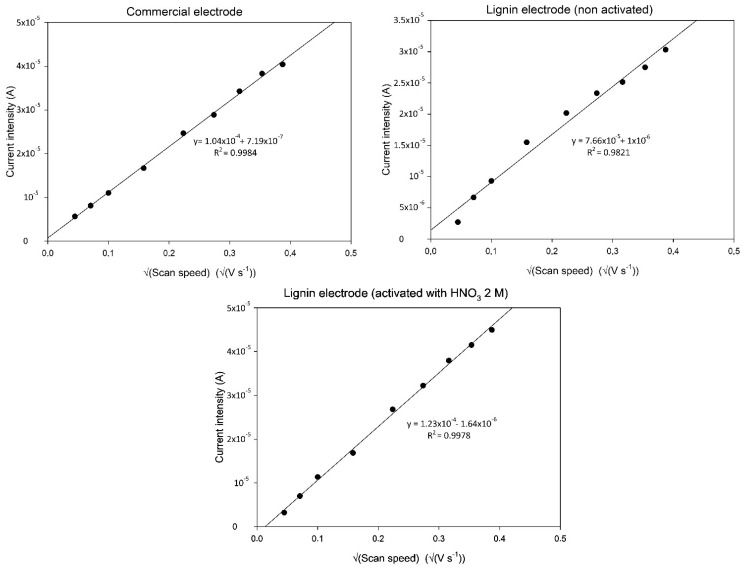
Correlation lines between the square root of cyclic voltammetry scan speed and current intensity of the anodic peak. Scan speeds tested: 2, 5, 10, 25, 50, 75, 100, 125,150 mV s^−1^; measurements performed using 50 µL of a solution of 1 mM K_3_Fe(CN)_6_ + 0.1 M KCl. Each data point is calculated by averaging three measurements performed on three electrodes each (total of nine measurements per data point).

Cyclic voltammetry scans were performed using the SPEs produced with commercial ink and with carbonized lignin (with or without HNO_3_ activation). The obtained correlation lines are reported in Figure 7.

As depicted in Figure 7, a good correlation was obtained for all three types of electrodes. The electrochemically active areas calculated for each type of electrode show how the non-activated carbonized lignin-based electrodes had an electroactive area of 0.1041 cm^2^, about 27% less than that of the electrodes produced using commercial ink (which had an electroactive area of 0.1416 cm^2^) and having the same WE diameter. This situation is reversed when considering the carbonized lignin-based electrodes activated using 2 M HNO_3_ as described in Section 2.7. Here, an electroactive area of 0.1813 cm^2^ was calculated, a marked increase compared to the commercial electrode (28% more) and the non-activated carbonized lignin-based electrode (74% more). These results suggest that the activation procedure using 2 M HNO_3_ affects the structure and superficial organization of the carbonized lignin fibers, making them more conductive and/or more capable of electron exchange with the solution containing the electrochemically active analyte.

Since the electrodes described in this work employ cellulose acetate to bind the conductive particles of the ink to the electrode surface, two tests were designed to test their resiliency when exposed to water-containing solutions.

In the first test, repeated CV scans were performed using the solution and parameters described in Section 2.8, employing both the commercial and carbonized lignin-based electrodes. Up to 100 consecutive scans were performed in a container with controlled humidity, to prevent the evaporation of the electrochemical mediator solution. The curves obtained during the 100th CV cycle with both types of electrodes were almost identical to the ones obtained in the initial cycles (data not shown), proving that the carbonized lignin-based electrodes have a stability in water solution under electrochemical stress comparable to that of the commercial electrodes.

A second test was designed to ascertain the stability of carbonized lignin-based electrodes after prolonged exposure to aqueous solutions: cyclic voltammetry of three electrodes produced using carbonized lignin ink was performed, after activation with 2 M HNO_3_, following the parameters described in Section 2.8. After the measurements, the electrodes were thoroughly washed and stored in bi-distilled water for one week. The electrodes were then dried and tested with CV measurements using the same solution and experimental conditions. The CV curves of each electrode overlap almost perfectly with the ones obtained before storage in water (data not shown), proving that the ink, even employing cellulose acetate as the binder, is suitable for long-term measurements in aqueous media.

### 3.8. Stretch Effect on Electrochemical Performances

To test the effect of the mechanical stress of stretching on the electrochemical performances of the electrodes, CV scans were performed on the carbonized lignin-based electrodes after different levels of stretching were applied to them. The tested stretch values were the same as the ones tested in Section 3.5, to see if a correlation emerged between the measured resistance and electrochemical performances. The results obtained, in terms of the recorded potential and intensity of the anodic and cathodic peaks, are reported in Table 3.

As can be seen, a higher stretching stress applied to the stretchable electrodes corresponds to a decrease of the current intensity responses and a shift of both the anodic and cathodic peaks to higher and lower potential values, respectively. Furthermore, higher stretch stresses correspond to lower measurement reproducibility (as can be seen from the increasing standard deviation reported).

### 3.9. Carbonized Lignin SPE Test as a Sensor Platform

**Figure 8:**
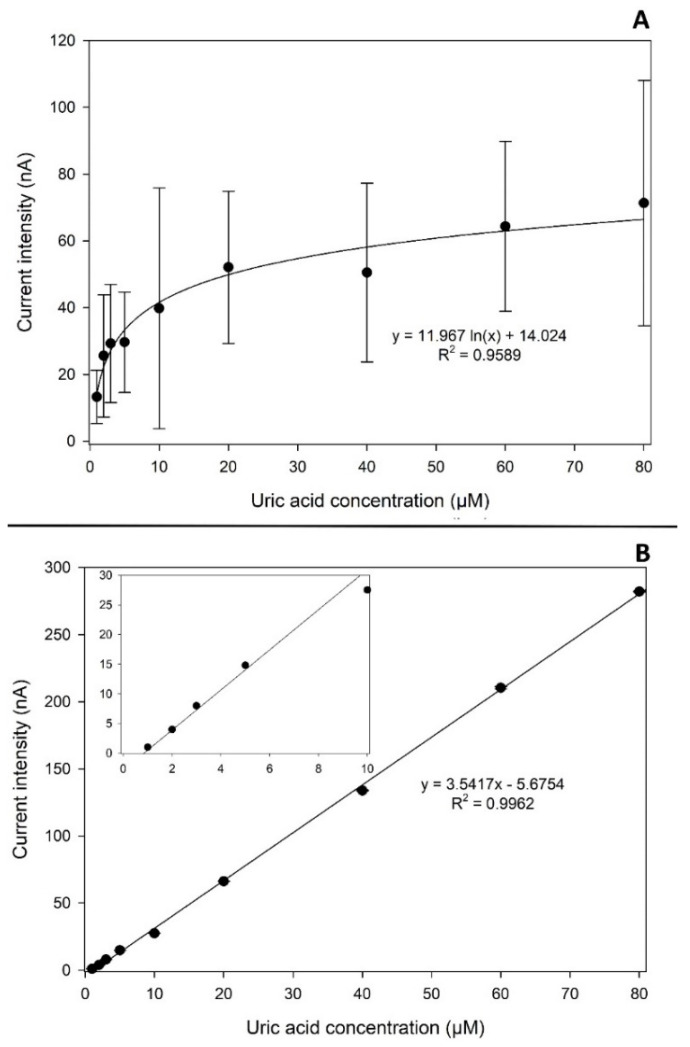
Calibration curve for uric acid performed using (**A**) electrodes produced with commercial carbon ink and (**B**) carbonized lignin-based SPE for concentrations of uric acid ranging from 1 µM to 80 µM. Measurements were performed with the chronoamperometry technique, with an applied potential of +0.2 V in PBS. The results shown are the average of three independent measurements.

As shown in Section 3.6, the developed carbonized lignin electrodes respond with good electrochemical performances when in the presence of a standard inorganic electroactive substance. Since the carbon material used for the printing has a very porous structure even after printing (as shown in Section 3.4), the electrode needed to be tested against an organic electroactive molecule to evaluate the presence of any fouling of the surface due to the interaction between the carbon-based working electrode surface and the electrochemical products. Uric acid was chosen as the target electroactive organic molecule, as it is known from the literature [42] that it spontaneously oxidizes in aqueous media when a potential of 0.2 V or higher is applied, forming allantoin. This substance, thanks to its numerous electron attractor and electron donor groups, is a good candidate to see if the developed WE suffers from fouling due to the formation of stable interactions with electrochemical products which result in reduced electrochemical performances of the electrode. Chronoamperometric measurements were conducted using both electrodes produced with commercial carbon ink and the developed carbonized lignin SPE, measuring uric acid in the concentration range between 1 and 80 µM. The results obtained, and the corresponding calibration curves, are reported in Figure 8.

From what can be observed in Figure 8A, the electrodes produced using commercial carbon ink show linearity only for uric acid concentrations in the range of 1–5 µM, after which the curve assumes a logarithmic-like trend. It can be assumed that some kind of fouling process on the surface of the WE occurs, inhibiting further electrochemical reactions.

On the other hand, as shown in Figure 8B, the SPEs produced using carbonized lignin respond with a good degree of linearity for all the investigated concentrations ranging from 1 to 80 µM (R^2^ = 0.9962, n = 3), with a limit of detection (calculated as signal-to-noise ratio S/N = 3) of 0.3 µM. It can be supposed that the developed electrodes do not interact with the species produced during the electrochemical reaction tested, at least at an observable level. The obtained parameters for both the commercial and carbonized lignin-based electrodes are reported in Table 4.

### 3.10. Carbonized Lignin SPE Test as a Platform for Enzymes

**Figure 9:**
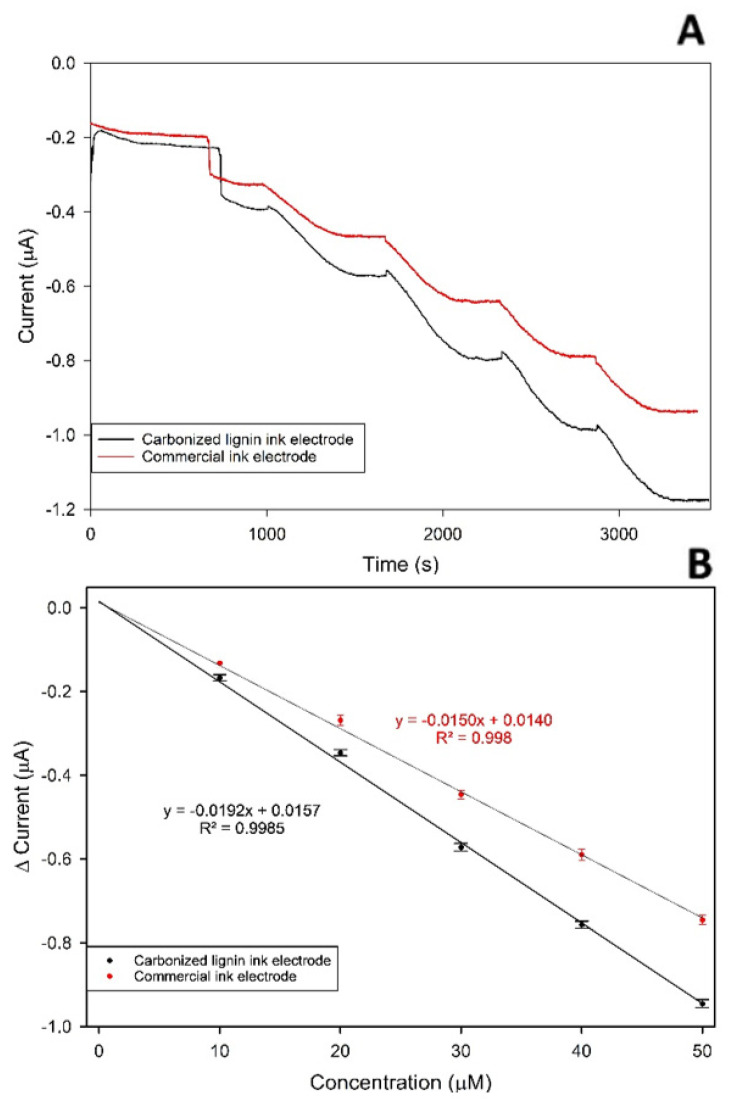
(**A**) Chronoamperogram after addition of increasing amounts of catechol using (black line) carbonized lignin SPE or (red line) commercial SPE with the laccase enzyme. Applied potential −0.16 V, sampling interval 1 s. (**B**) Calibration plot for the (black line) carbonized lignin SPE or (red line) commercial SPE with the laccase enzyme. Applied potential was −0.16 V (n = 3).

The laccase enzyme from *Trametes versicolor*, an enzyme that catalyzes the oxidation of catechol to 1,2 benzoquinone, was used since its reaction mechanisms are well known, and it has a simple electrode transmission towards the SPE on which it is deposited. To test the compatibility of the carbonized lignin SPE with biotransducers, the measurements were performed as follows: 1 unit of laccase was dissolved in 100 µL of acid citric/sodium citrate buffer (pH 4.5), and deposited on the SPE, covering the working, reference, and counter electrode. Chronoamperometry measurement was started with an applied potential of −0.16 V since at this potential the reaction catalyzed by laccase is detectable by electrochemical means according to the literature [43]. Once a stable baseline was obtained and recorded, a small volume of a concentrated solution of catechol was added to the drop on the SPE to obtain the desired catechol concentration in the range from 10 to 50 µM. After each catechol addition, the amperometric signal was recorded. The process was repeated for increasing concentrations of catechol, to evaluate the linearity of the response of the platform. The same procedure as described above was repeated using SPE produced using commercial ink as a comparison. An example of an obtained chronoamperometry measurement is reported in Figure 9A.

As shown in Figure 9B, the assembled system using carbonized lignin-based SPE showed remarkable linearity over the investigated concentration range of catechol (R^2^ = 0.9985 n = 3), with a limit of detection (calculated as signal-to-noise ratio S/N = 3) of 2.01 µM. On the other hand, the assembled system using the SPE produced using the commercial ink showed good linearity (R^2^ = 0.9980 n = 3), but a worse limit of detection (calculated as signal-to-noise ratio S/N = 3) of 5.00 µM. The obtained parameters for both the commercial and carbonized lignin-based electrodes are reported in Table 4.

## 4. Conclusions

In this work, a novel strategy was proposed to employ suitably modified lignin recovered from the waste products of the Eucalyptus tree paper industry, as a substitute for petroleum-derived compounds in the formulation of inks to produce screen-printed electrodes.

The lignin, previously subjected to a controlled carbonization process, was mixed with different organic solvents and binders to obtain a composition with both rheological and conductivity properties suitable for electrode creation. The combination that gave the best results was the one using cyclohexanone as a solvent to mix the carbonized lignin and cellulose acetate, each in a 5% *w/w* ratio.

The electrodes were printed both on flexible and stretchable substrates, demonstrating in both cases the excellent adhesion of the ink to the surface when under stress. Considering stretchable electrodes, the electrical resistance between each of the three electrodes including the SPE and the respective contact was also evaluated when the electrode was subjected to stretching stress and after repeated stress cycles. The results obtained underline how, for relatively small stretching (up to a 30% length increase compared to the original electrode length), resistance variations are small, even after repeated stress cycles.

Knowing from the reported SEM analysis that this carbonized lignin is composed of nano-dimensional fibers, different activation procedures were evaluated to increase the electrochemical response of the working electrode. The best results were obtained by activating the WE with a solution of 2 M HNO_3_ for 30 min, reaching electrochemical performances in cyclic voltammetry comparable with the ones obtained using structurally similar commercial electrodes.

Finally, to verify if the produced electrodes were suitable as sensors and compatible with biomediators for the development of biosensors, two tests were performed. In the first one, the SPE was used to assemble a sensor for uric acid detection, resulting in a linear response in the interval evaluated, with no apparent fouling of the employed electrodes. In the second test, the compatibility of carbonized lignin SPE with bioreceptors was tested using the laccase enzyme and catechol as the substrate. From the data obtained, it is evident that the assembled system responded very well in the interval evaluated, demonstrating that the electrodes described in this work could feasibly substitute those currently used based on petroleum-derived inks. The obtained values of the linear response range and limit of detection for the assembled biosensor are competitive with the values found in the literature for laccase biosensors [43,44,45,46]. In conclusion, the results obtained prove the suitability of the developed carbonized lignin SPEs as a sustainable and efficient support for the development of sensors and biosensors. The demonstrated properties lend support to the possibility of employing the developed carbonized lignin electrodes with different biorecognition elements [47] or transduction systems [48].

## Figures and Tables

**Table 1:** Resistance values, adhesion, and flexibility properties for tested inks on PET substrate after the heating process.

System	Resistance (Ω)	Flexibility	Coating Power
20% *w/w* CL/CA/CEx	415 ± 5	Good flexibility	Good adhesion
20% *w/w* CL/glycerol/CEx	2200 ± 450	Good flexibility	Bad adhesion
20% *w/w* CL/PU/CEx	1450 ± 270	Good flexibility	Good adhesion

**Table 2:** Resistance values measured for screen-printed electrode modified with inks having different concentrations of carbonized lignin.

Resistance (Ω)	5%	7.5%	10%	12%	15%
**WE-silver contact**	10 ± 1	11 ± 1	200 ± 50	55 ± 18	130 ± 60
**CE-SILVER CONTACT**	25 ± 2	34 ± 3	4000 ± 265	170 ± 75	840 ± 260

**Table 3:** Potential and current intensities obtained performing CV measurement with an electrode that underwent different levels of stretch stress. Each measurement was performed on three different electrodes; experimental parameters for CV are described in Section 2.8.

	No Stretch		10% Stretch		20% Stretch		30% Stretch		40% Stretch		50% Stretch	
	Potential (V)	Current (µA)	Potential (V)	Current (µA)	Potential (V)	Current (µA)	Potential (V)	Current (µA)	Potential (V)	Current (µA)	Potential (V)	Current (µA)
**Cathodic Peak**	0.046 ± 0.001	−69.162 ± 0.005	−0.015 ± 0.006	−60.548 ± 0.009	−0.087 ± 0.009	−58.715 ± 0.013	−0.103 ± 0.012	−52.793 ± 0.017	−0.158 ± 0.020	−44.695 ± 0.045	−0.196 ± 0.084	−40.030 ± 0.086
**ANODIC PEAK**	0.322 ± 0.002	52.430 ± 0.006	0.355 ± 0.004	49.683 ± 0.010	0.396 ± 0.007	47.628 ± 0.011	0.438 ± 0.014	42.915 ± 0.014	0.497 ± 0.059	36.549 ± 0.095	0.572 ± 0.097	30.451 ± 0.195

**Table 4:** Comparison of the electrochemical properties found for the commercial and carbonized lignin-based electrodes tested with uric acid as electroactive substance or the *Trametes versicolor* enzyme and catechol as substrate.

Type of Electrode	Limit of Detection		Limit of Linearity	
	Uric Acid	Laccase	Uric Acid	Laccase
**COMMERCIAL INK**	1.0 µM	2.01 µM	5.0 µM	50.00 µM
**CARBONIZED LIGNIN INK**	0.3 µM	5.00 µM	80.0 µM	50.00 µM

## Data Availability

Part of the data presented in this study are available on request from the corresponding author. The data are not publicly available due to “Non-disclosure agreements” between authors.
